# Metachronous Eosinophilic Granuloma of Rib in an Adult Patient

**DOI:** 10.7759/cureus.20670

**Published:** 2021-12-24

**Authors:** Dilvin Ozkan, Şevki M Demiroz, Muhammet Sayan, Merve Turan, İsmail C Kurul

**Affiliations:** 1 Thoracic Surgery, Gazi University, Ankara, TUR; 2 Thoracic Surgery, Gazi University Medical School, Ankara, TUR; 3 Pathology, Gazi University Medical School, Ankara, TUR; 4 Thoracic Surgery, Gazi University School of Medicine, Ankara, TUR

**Keywords:** surgical treatment, metachronous tumour, chest wall tumour, eosinophilic granuloma, langerhans cell histiocytosis

## Abstract

Eosinophilic granuloma (EG) is the unifocal osseous form of Langerhans cell histiocytosis (LCH), which usually affects the skull and long bones. Although it most commonly affects the pediatric age group, it can rarely be seen in adults. Skeletal involvement is common in adult patients, but isolated rib involvement is extremely rare. Differential diagnosis includes other osteolytic lesions such as Ewing’s sarcoma, tuberculosis, multiple myeloma, lymphoma, and primary bone malignancy.

The diagnosis must be confirmed histopathologically. In addition to pathological Langerhans cells, inflammatory cells such as lymphocytes, eosinophils, and macrophages are observed in microscopy. Immunohistochemically, CD1a, S-100, and Langerin positivity are observed in biopsy and/or surgical excision material.

Treatment options may vary depending on the localization and extent of the disease. In unifocal EG, close observation of the patient may be preferred, as well as surgical excision, radiotherapy, and intra-lesional steroid administration. The prognosis in patients with a single bone lesion is quite good compared to other groups.

In this case report, we present a metachronous EG of rib developed in two different ribs by an interval of seven years, which were both surgically treated. In this mild variant of LCH, surgical resection with clean margins has a favorable outcome without the need for additional adjuvant therapy. Metachronous tumors may develop in isolated unifocal bone EGs, and long-term follow-up is mandatory.

## Introduction

Langerhans cell histiocytosis (LCH) is an idiopathic disease that occurs as a result of the localized or widespread accumulation of Langerhans cells originating in the bone marrow in various tissues for an unknown reason [1]. Although it most commonly affects the pediatric age group, it can rarely be seen in adults. Annual incidence is 3-5/1.000.000 cases in the pediatric age group, while it is 1-2/1.000.000 cases in the adult group.

Eosinophilic granuloma (EG) is the unifocal osseous form of LCH, which usually affects the skull and long bones [2]. Skeletal involvement is common in adult patients, but isolated rib involvement is extremely rare, and it makes 6% of total cases reported [3]. In this case report, we present a metachronous EG of rib developed in two different ribs by an interval of seven years, which were both surgically treated.

## Case presentation

**Figure 1:**
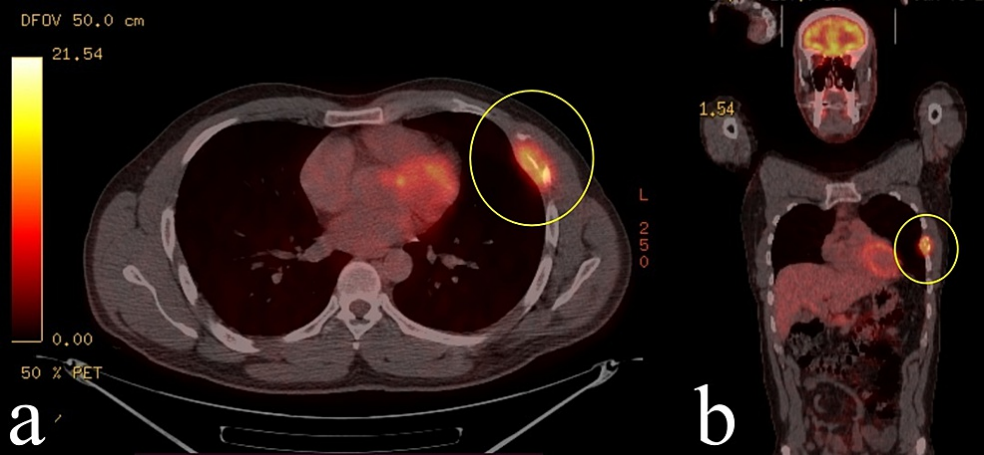
Eosinophilic granuloma involving the fifth and sixth ribs with pathologic 18F-FDG uptake (SUVmax: 6,8) on PET/CT axial (a) and coronal (b) images (yellow circle) The first admission was in 2014. FDG, Fluorodeoxyglucose; SUVmax, maximum standardized uptake value; PET/CT, positron emission tomography/computed tomography.

**Figure 2:**
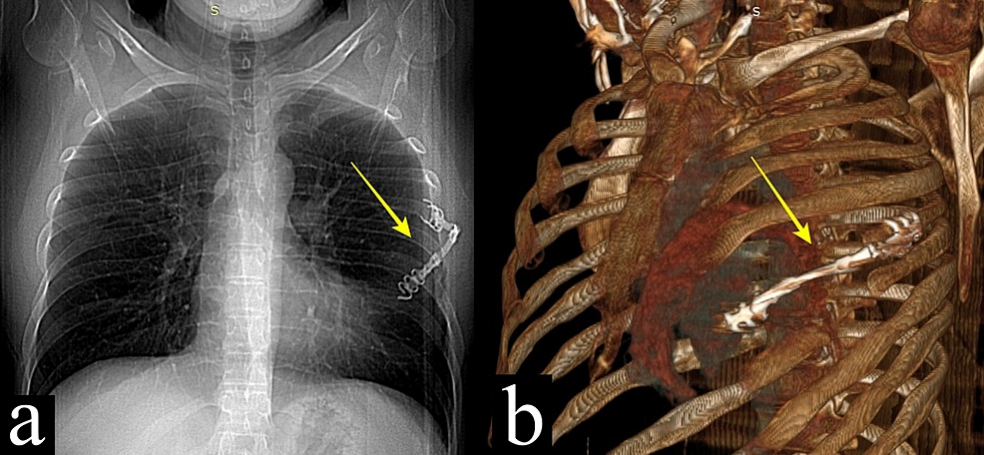
Postoperative chest x-ray (a) and 3D reconstruction of computed tomography (b) showing titanium bar reconstruction of the resected fifth rib (yellow arrow)

**Figure 3:**
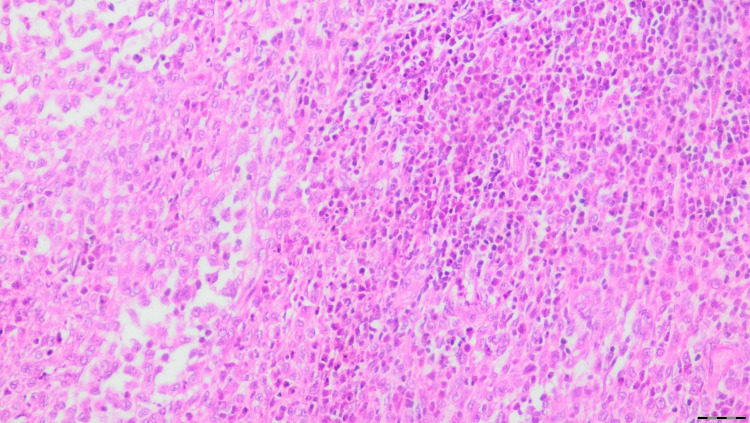
Result of the histopathological examination The tumor consists of cells with prominent grooved nuclei, delicate chromatin, and moderate amounts of eosinophilic cytoplasm (HE, x20).

Informed consent was obtained from the patient for publication of this case. A 39-year-old male patient, admitted with chest pain in 2014, was found to have an expansile lesion in the left fifth rib in his radiological examinations, and surgical resection was decided (Figure 1). Partial resection of the left fifth to the sixth rib was performed and was reconstructed with a titanium bar (Figure 2). The result of the histopathological examination was reported as LCH (Figure 3). Surgical margins were clear of tumor. The patient did not receive any adjuvant therapy after the surgery and was not available for follow-up.

**Figure 4:**
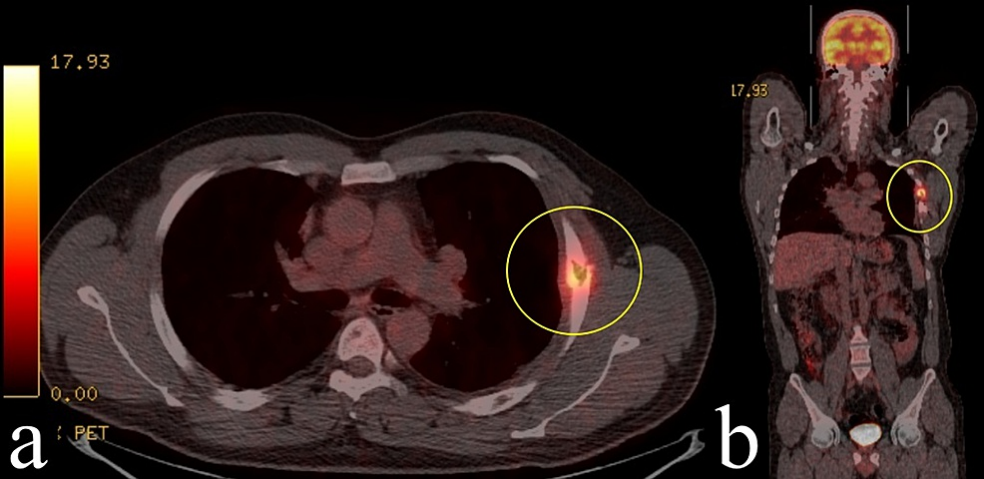
Osteolytic lesion on the left fourth rib (yellow circle) with pathologic uptake (SUVmax: 6,5) on PET/CT axial (a) and coronal (b) images The second admission was in 2021. SUVmax, Maximum standardized uptake value; PET/CT, positron emission tomography/computed tomography.

**Figure 5:**
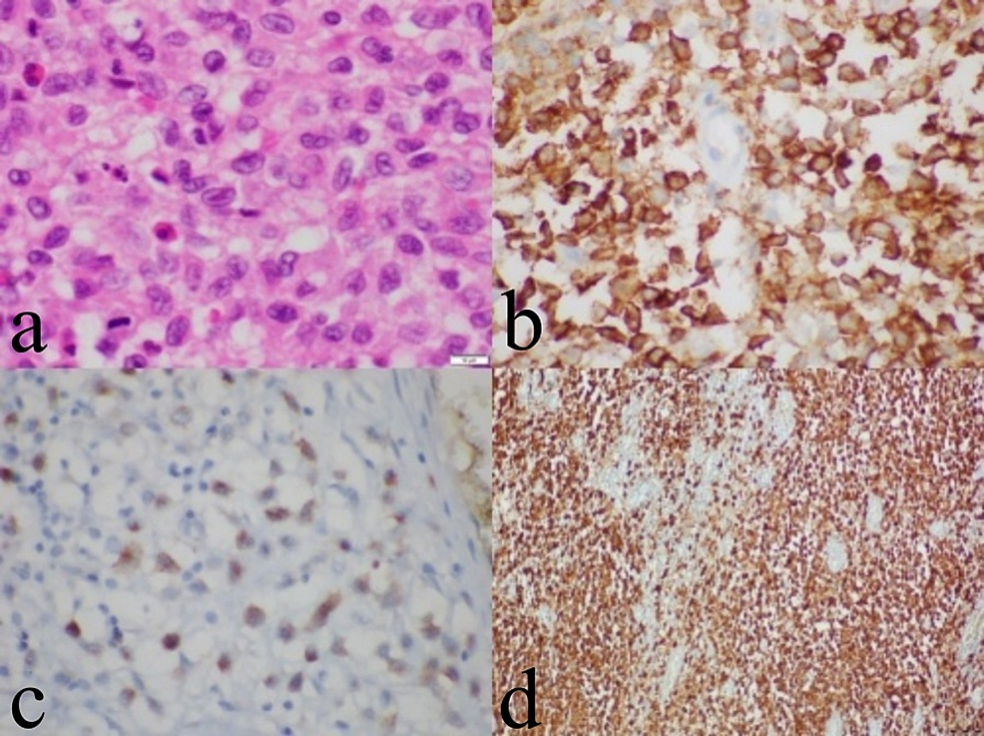
The final histopathological examination On a background accompanied by eosinophilic leukocytes, the tumoral development consisting of cells with large eosinophilic cytoplasm, nuclear notch, and vesicular nuclei with local nucleoli prominences was observed (HE, x1000) (a). In the immunohistochemical study, tumor cells were positive with CD1a (b), Langerin (c), and S100 (d).

In 2021, seven years after the first operation, the patient was admitted again with the complaint of increasing pain that started after a blunt trauma to the left chest wall. On thorax computed tomography (CT), an expansile hypodense lesion of approximately 3 cm x 2 cm was observed in the lateral arc of the left fourth rib. F-18 fluorodeoxyglucose (FDG) positron emission tomography/computed tomography (PET/CT) showed pathological uptake (SUVmax: 6,5) in the lytic lesion in the left fourth rib and also changes secondary to the previous operation in the anterolateral arcs of the left fifth to sixth ribs (Figure 4). Surgical resection was decided for the patient. On exploration, an approximately 3-cm off-white lesion with a smooth surface was observed on the left fourth rib. Extrapleural partial resection of the fourth rib was performed. The final histopathological examination revealed LCH (Figure 5). Microscopically, the surgical margins were tumor-negative. Immunohistochemically, the tumor cells were positive for S-100 and CD1a and weak positive for Langerin (CD207). Our biopsy was decalcified in %10 formic acid, and RT-PCR was unable to identify BRAF mutations probably because of DNA damage caused by the acid although LCH usually has to activate mitogen-activated protein kinase (MAPK) pathway mutations, especially BRAF V600E.

The patient was discharged without any problems on the second day after the operation. The patient was evaluated by the multidisciplinary council; no additional treatment was required, and close follow-up was recommended. The patient was doing well in his second month of follow-up.

## Discussion

LCH is a disease characterized by monoclonal proliferation of Langerhans cells of bone marrow origin; the exact etiology is unknown. Symptoms vary according to the localization and affected organ. The annual incidence was reported as 2-9/1.000.000 cases in the pediatric group (under the age of 15). But it is seen rarer in adults (1-2/1.000.000 cases per year) [3,4].

EG, which is the mildest variant and localized form of LCH, may present in two ways: a single bone lesion (monostotic) or multiple bone lesions (polyostotic). Skeletal involvement is more common in men. The most frequent site is mandible (30%) followed by cranium (21%), vertebrae (13%), and extremities (13%). While the rib involvement is rare (6%), it is more frequent in adults than in the pediatric population. In a report by Oh et al. including 211 adults and 330 pediatric patients, the rib involvement ratio was 25% for adults and 12% for the pediatric group [3].

Isolated rib involvement is extremely rare, and its diagnosis is challenging. Differential diagnosis includes other osteolytic lesions such as Ewing’s sarcoma, tuberculosis, multiple myeloma, lymphoma, and primary bone malignancy [3,4]. Thus, there are no defined specific findings; a definite diagnose cannot be achieved by only imaging studies. While the first step in imaging is x-ray, CT, MRI, and scintigraphy may be required. F-18 PET/CT is used to rule out a systemic variant of LCH [3,5]. The diagnosis must be confirmed by histopathological examination. In addition to pathological Langerhans cells, inflammatory cells such as lymphocytes, eosinophils, and macrophages are observed in microscopy. Immunohistochemically, CD1a, S-100, and Langerin positivity are observed in biopsy and/or surgical excision material.

There is no standard treatment for LCH. Treatment options may vary depending on the localization and extent of the disease. In unifocal EG, close observation of the patient may be preferred, as well as surgical excision, radiotherapy, and intra-lesional steroid administration. Systemic chemotherapy is recommended in multisystem involvement [4]. The prognosis in patients with a single bone lesion is quite good compared to other groups [6]. In a study including 61 patients diagnosed with Langerhans cell histiocytosis with single focal bone involvement, recurrence was seen in only two patients after surgical resection [7]. In another study, four-year survival was 90% or more in patients with single bone involvement [1]. In the present case, both in the years 2014 and 2021, there was no sign of systemic disease. So multidisciplinary council did not advise any further treatment.

## Conclusions

In conclusion, we present an extremely rare metachronous rib EG in an adult patient presenting with an interval of seven years. In this mild variant of LCH, surgical resection with clear margins has a favorable outcome without the need for additional adjuvant therapy. Metachronous tumors may develop in isolated unifocal bone EGs, and long-term follow-up is mandatory.
